# Vancomycin-specific Urinary Immunostaining for Noninvasive Screening of Vancomycin-associated Cast Nephropathy

**DOI:** 10.1016/j.xkme.2025.101188

**Published:** 2025-11-19

**Authors:** Cédric Rafat, Ellie Tang, Jeremy Zavorski, David Rozenblat, Lara Zafrani, Remi Chieze, Stéphane Gaudry, Yosu Luque, Emmanuel Letavernier

**Affiliations:** 1 Soins Intensifs Néphrologiques et Rein Aigu, Tenon Hospital, AP-HP, Paris, France; 2 Explorations fonctionnelles multidisciplinaires, Hôpital Tenon, AP-HP, Paris, France; 3 Sorbonne Université, Inserm, Common and Rare Kidney Diseases: from Molecular Events to Precision Medicine, CoRaKiD, F-75020 Paris, France; 4 Department of Medical Intensive Care Unit, Saint-Louis Hospital, AP-HP, Paris, France; 5 Unité INSERM U944, Saint-Louis Research Institute, Paris, France; 6 Université de Paris Cité, Paris, France; 7 Department of Medical Intensive Care Unit, Avicenne Hospital, AP-HP, Bobigny, France; 8 Université Paris Sorbonne Paris Nord, Bobigny, France

To the Editor:

Among drugs acknowledged for their nephrotoxicity, vancomycin stands out both as being a potent cause of acute kidney injury (AKI) and as one of the most universally prescribed antimicrobial drugs, especially in intensive care units. In case of drug supratherapeutic dose, studies have recently shown that vancomycin can precipitate with uromodulin causing nanospheric obstructive aggregates in the tubular lumen in experimental models and in patients.^1^, ^2^, ^3^ Further exploration of this mechanism has been limited by the need for kidney tissue to perform immunostaining.

Urine-based vancomycin immunostaining may offer a noninvasive method to diagnose vancomycin-associated acute kidney injury (V-AKI).^4^ However, it remains unclear whether vancomycin casts are specific to V-AKI or simply reflect vancomycin accumulation in the urine. This study aimed to assess the association between vancomycin immunostaining-positive casts and V-AKI occurrence.

For this purpose, we conducted a retrospective, observational study of the urinalysis of patients exposed to vancomycin. A total of 28 patients who had received vancomycin were recruited from 4 different intensive care units and included in the study. Twelve patients were classified as cases (V-AKI), whereas 16 patients were assigned to the control group based on the Naranjo adverse drug reaction probability scale.

In the V-AKI group, only 2 patients had preexisting chronic kidney disease (Table 1). All patients developed Kidney Disease: Improving Global Outcomes stage III AKI, and 2 (16.7%) required renal replacement therapy. Similarly, 8 patients (75%) were exposed to other nephrotoxic drugs. None of the patients with V-AKI had presumed intrinsic kidney disease. Concomitant factors contributing to AKI are shown in Table 1. The median daily dose and cumulative dose were 1 g (0.8; 1.13) and 3 g (2; 4), respectively. In 8 cases (75%), patients experienced vancomycin supratherapeutic dose with a median peak through level of 60 mg/L (41; 72). All patients in the V-AKI group yielded positive urine immunostaining for vancomycin. Vancomycin-positive casts were equally divided into granular casts (n = 6) and smooth cylindrical casts (n = 6). At 3 months of follow-up, all patients had recovered their baseline kidney function. The control group (Table 2) consisted of patients with normal baseline kidney function who did not develop AKI (n = 7), patients with stage V chronic kidney disease (CKD, n = 5), and patients who developed Kidney Disease: Improving Global Outcomes stage II/III AKI during vancomycin exposure but whose AKI was probably not related to the introduction of vancomycin (n = 4). Control patients received a median daily dose and cumulative dose of 1.7 g (0.9; 2.5) and 7.4 g (3.8; 10.5), respectively.

**Table 1:** Patients With a Strong Suspicion of Vancomycin-Induced Cast Nephropathy

Age, sex	62/M	31/M	66/M	59/M	49/M	70/F	70/F	38/M	57/M	67/M	78/F	60/F
Body mass index	27	24	43	41	22	51	44	42	31	20	27	31
Baseline creatinine (μmol/L) and eGFR (mL/min)	73/101	80/77	77/98	240/25	97/79	60/110	79/85	94/79	85/82	49/125	58/118	94/59 mL/min
Comorbid conditions	None	BVH and alcohol-associated cirrhosis	D2	HTN, D2, HTN-related cardiopathy	AIDS	None	CLL Neutropenic colitis	High-grade B cell NHL	Mantle cell NHL Neutropenic colitis	D2 LV dysfunction	Metastatic prostate cancer	D2, KT
AKI staging (KDIGO)/peak plasma creatinine (μmol/L)	3/570	3/652	3/NA	3/507	3/NA	3/NA	3/389	3/434	3/340	3/460	3/350	2/280
RRT requirement	No	No	Yes : 10 days	No	Yes : 10 days	Yes : 15 days	No	No	No	No	No	No
Indication for vancomycin therapy	*E. faecium* and *P. aeruginosa* MVAP	*S. aureus* BSI	Polymicrobial osteitis	Undocumented dermo-hypodermititis	*E. faecium* Angiocholitis	*S. aureus* Surgical site infection	*E. faecium* UTI	*S. epidermidis* CIA	*S. haemolyticus* CIA and BSI	*S. aureus* mediastinitis	*S. aureus* UTI and BSI	Breast abscess Septic shock
Vancomycin dose (daily dose/therapy duration)	2.5 g/2 days	4 g/2 days?^a^	4 g/11 days	1 g/1 day	1.5 g/5 days	4 g/5 days	2 g/2 days	7 g/8 days	5 g/2 days	NA	3 g/ 3 days§	2 g/2days
Peak vancomycin through level (mg/L)^b^	41	72	NR	NR	27	122	61	60	18	83	NR	43
Competing cause of AKI	No	No	No	No	Severe septic shock	No	Hypovolemia	No	Hypovolemia	LV dysfunction with cardio-renal syndrome	No	Septic shock
Associated nephrotoxic therapy	Piperacillin-Tazobactam	None	ICM	ICM	AminoG Aciclovir	AminoG	None	None	AminoG	AminoG ICM	None	AminoG Tacrolimus
Vasopressor support (drug/peak dose)	No	No	No	No	Yes	No	No	No	No	No	No	Yes
Delay vancomycin initiation-urinary microscopy	6 days	7 days	25 days	6 days	5 days	5 days	3 days	11 days	3 days	14 days	10 days	2 days
Urine pH	6.2	5.6	5.3	5.9	NR	8.2	5.3	5.3	5.3	5.5	5	6
Vancomycin mode of administration (continuous vs discontinuous)	D	C	C	D	D	C	D	C	C	D	C/D^c^	C
Morphology of vancomycin casts	Smooth cylinder	Smooth cylinder	Granular	Smooth cylinder	Smooth cylinder	Smooth cylinder	Granular	Smooth cylinder	Granular	Granular	Granular	Granular
Vancomycin-specific urine immunostaining	Positive	Positive	Positive	Positive	Positive	Positive	Positive	Positive	Positive	Positive	Positive	Positive
Naranjo score	8	8	7	7	5	8	5	8	5	5	8	5
Renal function course Creatinine μmol/L /eGFR mL/min	6-month FU: 110/50	1-month FU: 85/75	2-month FU: 115/51	5-month FU: 260 /23	3-month FU: 110/62	3-month FU: 50 /102	1-month FU: 98 /52	8-month FU: 84 /88^a^	1-month FU: 122 /58	3-month FU: 100/50	3-month FU: 52/119	3-month FU: 90 /60

Abbreviations: AIDS, Acquired Immunodeficiency Syndrome; aminoG, aminoglycoside; BVH, chronic Hepatitis B; BSI, bloodstream infection; CIA, chemotherapy-induced aplasia; C, continuous; CLL, chronic lymphocytic leukemia; D, discontinuous ; D2, type 2 diabetes; F, female; FU, follow-up; HTN, arterial hypertension; ICM, intravascular contrast media; LV, left ventricle; M, male; MVP, mechanical-ventilation–acquired pneumonia; NA, not applicable; NE, norepinephrine; NHL, non-Hodgkin lymphoma; UTI, urinary tract infection.

a Aminoglycoside overdosing.

b Supratherapeutic dose was defined as at least one occurrence of vancomycin plasma supratherapeutic dose, determined as at least one occurrence of plasma through levels > 20 mg/L and steady state levels > 25 mg/L in patients who received intermittent infusion or continuous administration, respectively.

c The 24-hour vancomycin dose was administered over a 6-hour period.

**Table 2:** Patients Exposed to Vancomycin Without or With a Low Suspicion of Vancomycin-Induced Cast Nephropathy

Age, sex	M, 62	F, 84	M, 40	M, 73	M, 64	M, 71	M, 61	M, 51	F, 21	M, 43	F, 66	M, 74	M, 82	F,69	M,70	M,63
Body mass index	33	27	20	38	32	21	27	28	19	21	39	21	28	30	21	26
Preexisting CKD	No	No	No	No	No	No	No	Stage V	Stage V	Stage V	Stage V	Stage V	Stage III	Stage III	Stage II	No
Kidney function status/creatinine (μmol/L)/eGFR (mL/min) Prior initiation of vancomycin	N 85/62	N 47/91	N 60/120	N 67/95	N 57 /105	N 74/93	N 62/ 20	Stage V 736/7	Stage V 1028/4	Stage V 355/14 (post HD)	Stage V 295/15	Stage V 854/6	KDIGO 3 AKI^a^ 297/18	KDIGO 3 AKI^a^ 453/9	KDIGO 2 AKI 145/45	KDIGO 2 AKI 126/55
Comorbid conditions	AML	HTN, D2	Acute pancreatitis	HTN, dyslipidemia, vascular disease	Rectum cancer	Liver cancer, biliary prothesis, D2	AML with allo-SCT, HTN, BHV	HTN, D1	IgAN	HTN	NSCC, MN	HTN, KT	HTN, D2	D2, alcohol abuse, L/KT	RPF, KT, NODAT	D2, pancreas cancer
Kidney function course within 7 days following exposure to vancomycin	Unchanged	Unchanged	Unchanged	Unchanged	Unchanged	Unchanged	Unchanged	Unchanged	Unchanged	NA started on HD	Unchanged	Unchanged	Improved	Improved	Improved	Improved
RRT requirement during hospitalization, following exposure to vancomycin	No	No	No	No	No	No	No	Chronic PD	Chronic HD	Chronic HD	No	No	No	No	No	No
Indication for vancomycin therapy	*E*. *faecium* Primary BSI	Undocumented Surgical site infection	*E. avium* Surgical site infection	*E*. *avium* Surgical site infection	Undocumented Peritonitis	*E*. *faecium* Angiocholitis	*E*. *faecium* BSI	Undocumented PD-related peritonitis	Undocumented SSTI	C. spp CRI	*C*. *striatum* CRI	Undocumented AV fistula infection	Undocumented Respiratory tract infection	*E*. *faecium* KT-related UTI	*E*. *faecium* KT-related UTI	*E*. *faecium* Angiocholitis
Vancomycin dose (daily dose/therapy duration)	15 g/5 days	10 g/3 days	2 g/ 7 days	7.8 g/3 days	12 g/3 days	4 g/4 days	25 g/10 days	1 g/2 days	7 g/7 days	3.3 g/4 days	16.5 g/9 days	2 g/1 day	10 g/5 days	6.2 g/4 days	6.75 g/8 days	7.8 g/ 6 days
Vancomycin mode of administration (continuous vs discontinuous)	D	C	C	C	C	C	C	D	D	D	D	D	D	D	C	C
Peak vancomycin through level (mg/L)^b^	26	37	29	39	31	50	24	15	62	25	50	NP	24	42	51	33.5
Competing cause of AKI	No	Septic shock	Septic shock	Septic shock	No	No	No	No	Moderate LVD	No	No	No	Septic shock^c^	Septic shock^c^	KT-related UTI	Cholemic AKI
Associated nephrotoxic therapy	No	aminoG	ICM, aminoG	ICM	aminoG	aminoG	ciclosporin	No	No	No	No	tacrolimus	No	tacrolimus	No	No
Vasopressor support (drug/peak dose)	No	Yes	Yes	Yes	No	No	No	No	No	No	No	No	No	No	No	No
Casts/crystals on urine microscopy	Granular	No	Granular/ACCP^d^	No/ACCP^d^	No	No	No	No	No	No	No	No	No	No	NA	No
Vancomycin-specific urine immunostaining	Negative	Negative	Negative	Negative	Negative	Negative	Negative	Negative	Negative	Negative	Negative	Negative	Negative	Negative	Negative	Negative
Naranjo score	NA	NA	NA	NA	NA	NA	NA	NA	NA	NA	NA	NA	2	2	1	3

Color codes: coral, normal baseline kidney function and no AKI; yellow, CKD and no AKI; peach: AKI with preexisting CKD; crimson: AKI with normal prior baseline kidney function.

Abbreviations: AKI, acute kidney injury; AML, acute myeloid leukemia; AV, arteriovenous; BSI, bloodstream infection; BVH, chronic hepatitis B; CKD, chronic kidney disease; CRI, catheter-related infection; D1, type 1 diabetes; F, female; HD, hemodialysis; HTN, arterial hypertension; ICM, intravascular contrast media; IgAN, IgA nephropathy; KT, kidney transplantation; L/KT, combined liver and kidney transplantation; LVD, left ventricular dysfunction; M, male; NA, not applicable; NE, norepinephrine; N, normal; NODAT, new onset diabetes after transplantation; NSCC, non-small cell carcinoma; PD, peritoneal dialysis; RPF, retroperitoneal fibrosis; RRT, renal replacement therapy; SCT, stem cell transplantation; SSTI, skin and soft tissue infection; UTI, urinary tract infection.

^a^ Vancomycin therapy was initiated while kidney function was improving without altering the course of its recovery.

^b^ Supratherapeutic dose was defined as at least one occurrence of vancomycin plasma supratherapeutic dose, determined as at least one occurrence of plasma through levels > 20 mg/L and steady state levels > 25 mg/L in patients who received intermittent infusion or continuous administration, respectively.

^c^ Patient weaned from vasopressor support when vancomycin was initiated.

^d^ Amorphous carbonated calcium.

Because all patients with a Naranjo score ≥5 showed positive vancomycin urine immunostaining, but no such casts were observed in patients with a low Naranjo score, the sensitivity and specificity of vancomycin staining were both determined to be 100%. The median BMI of patients in the case group was 31 (21; 41.3) compared with 27 (21; 30.5) in the control group (*P* = 0.06). The median time from initiation of vancomycin to urinalysis did not differ between the case and control groups with values of 6 days (4.5; 10.3) and 5.5 days (2.75; 16), respectively (*P* = 0.44). Peak vancomycin levels were greater in the case group compared with the control group (*P* = 0.052). Compared with the case group, control patients were exposed to a higher cumulative dose of vancomycin (*P* = 0.01), but the daily dose of vancomycin did not differ significantly (*P* = 0.1). The mode of vancomycin administration (continuous vs discontinuous) showed no significant difference between groups (*P* = 1).

In this retrospective study, vancomycin urine immunostaining yielded excellent sensitivity and specificity for identifying patients with a strong suspicion of V-AKI. Our findings refute the hypothesis that positive urine vancomycin immunostaining merely reflects passive vancomycin filtration and/or excretion in urine regardless of the presence of AKI or overexposure to vancomycin. Controls were carefully selected based on the Naranjo score and included individuals from various clinical contexts, including AKI, CKD, and/or vancomycin supratherapeutic dose, to validate vancomycin urine immunostaining results.^5^ Patients with vancomycin-positive urine immunostaining exhibited a distinct and consistent clinical course, marked by severe AKI, followed by a favorable renal outcome, with all patients recovering their baseline renal function. This group of patients also tended to display a higher body mass index and higher vancomycin peak through levels.^6^ Although not statistically significant, these results suggest a scenario in which higher body mass index predisposes patients to vancomycin supratherapeutic dose, potentially triggering V-AKI.

Kidney biopsies were not carried out in any of the patients, so the results of the urine samples could not be compared with histological evidence. Consequently, other potential causes of kidney injury may have been discounted, and V-AKI may have been overdiagnosed. Urine sampling for microscopic examination relied primarily on ad hoc decisions made by the attending physician in consultation with a nephrologist. Repeat urinary sampling was not provisioned for, so vancomycin-positive casts may have been overlooked.

Despite these limitations and small cohort size, these preliminary results highlight the potential of vancomycin-positive immunostaining to identify patients with V-AKI who may achieve a favorable renal outcome even after severe AKI.

## Supplementary Materials

Supplementary File (PDF) Item S1, Figures S1-S2
