# Supplementary material for: Vancomycin-specific Urinary Immunostaining for Noninvasive Screening of Vancomycin-associated Cast Nephropathy
Source: Kidney Med. 2025 Nov 19;8(2):101188. doi: 10.1016/j.xkme.2025.101188 (PMC12835420; doi:10.1016/j.xkme.2025.101188)
Supplement: Supplementary File (PDF) — Item S1, Figures S1-S2 [file mmc1.pdf]

Item S1. Detailed methods

The results of light microscopic examination of urine samples and the clinical data of patients exposed to vancomycin were collected retrospectively between January 2021 and May 2024 at Tenon hospital using an excel spreadsheet. The clinical records and microscopic examination results were retrieved retrospectively and analyzed using the database of the kidney physiology center which carries out over 3300 routine urinalysis per year. This study was conducted in accordance with declaration No. 004 of the CNIL (National Commission for Data Protection and Liberties) for data protection and registered accordingly (reference 2232992). It was designed as an exploratory, proof-of-concept investigation in line with STROBE guidelines. Vancomycin dosing, including the timing of plasma level measurements, was left to the discretion of the treating physicians. Patients were assigned to the case group defined by V-AKI criteria when1) they developed AKI following exposure to vancomycin 2) they met the "probable" causality (Naranjo score ≥ 5) or "certain" causality (Naranjo score > 9) score. In all other cases, patients exposed to vancomycin were allocated to the control group. Within this group, patients were classified as follows: 1) patients without acute kidney injury (AKI) or chronic kidney disease (CKD); 2) patients with CKD whose kidney function remained unchanged after vancomycin exposure; and 3) those who developed AKI after vancomycin exposure, with normal baseline kidney function or CKD, but whose Naranjo score indicated a 'doubtful' association (≤ 3). Vancomycin supratherapeutic dose was defined as at least one occurrence of vancomycin plasma supratherapeutic dose, determined as at least one occurrence of plasma through levels > 20 mg/L (5) and steady state levels > 25 mg/L in patients who received intermittent infusion or continuous administration, respectively. Examination of the urine samples for vancomycin cast detection and immunostaining has been described elsewhere (see supplementary appendix). Briefly, a pellet of urine was spread on a slide and immunostained with a specific anti-vancomycin antibody (Abbot 6E-4421, 1/1000) prior to microscopic examination (see below).

Values were expressed as medians with interquartile ranges (IQRs), and comparisons between groups were performed using the Mann-Whitney U test. Ordinal variables were compared using the Chi-square test or Fisher's exact test, as appropriate, based on the distribution of the data.

Figure S1: Vancomycin urinary immunostaining

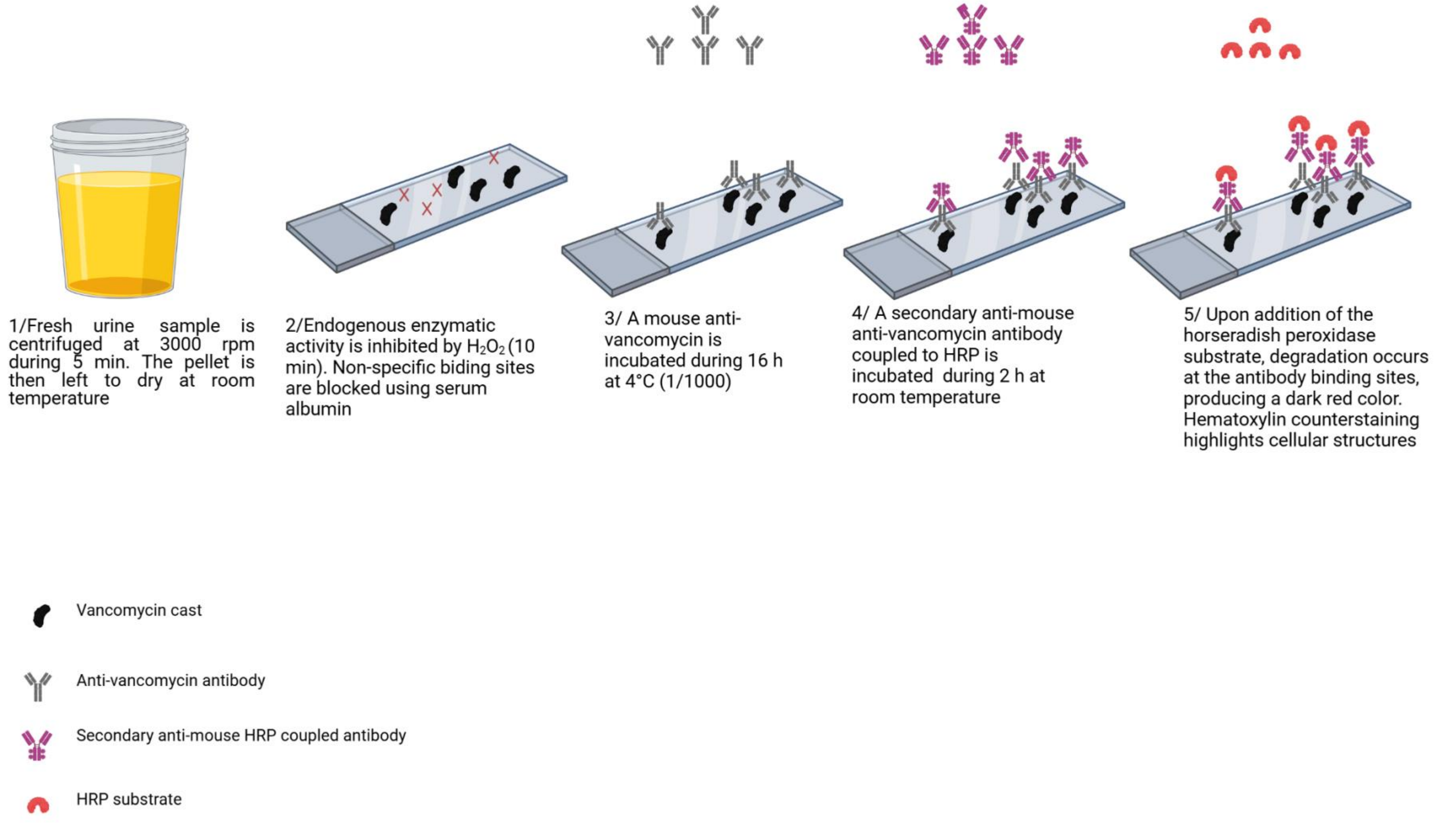

Figure S2: microscopic images of positive urinary vancomycin-specific immunostaining

2a

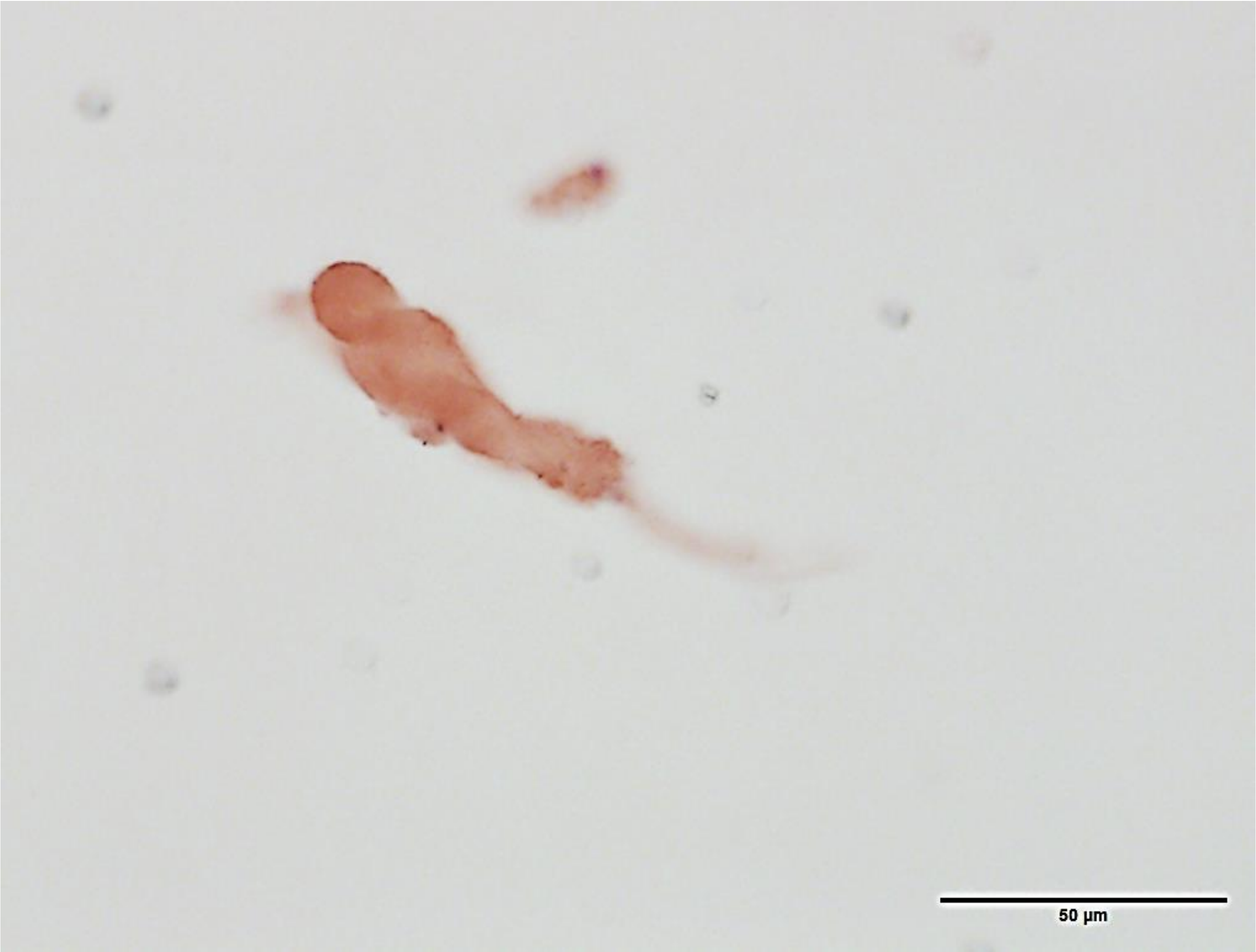

2a) Optical microscopy: Urine sample obtained from a 59-year-old male patient who received 1 g of vancomycin over 24 hours, developed stage III acute kidney injury (AKI), and exhibited smooth vancomycin-specific casts. Peak vancomycin through level: note recorded. Urinary pH: 5.9

2b

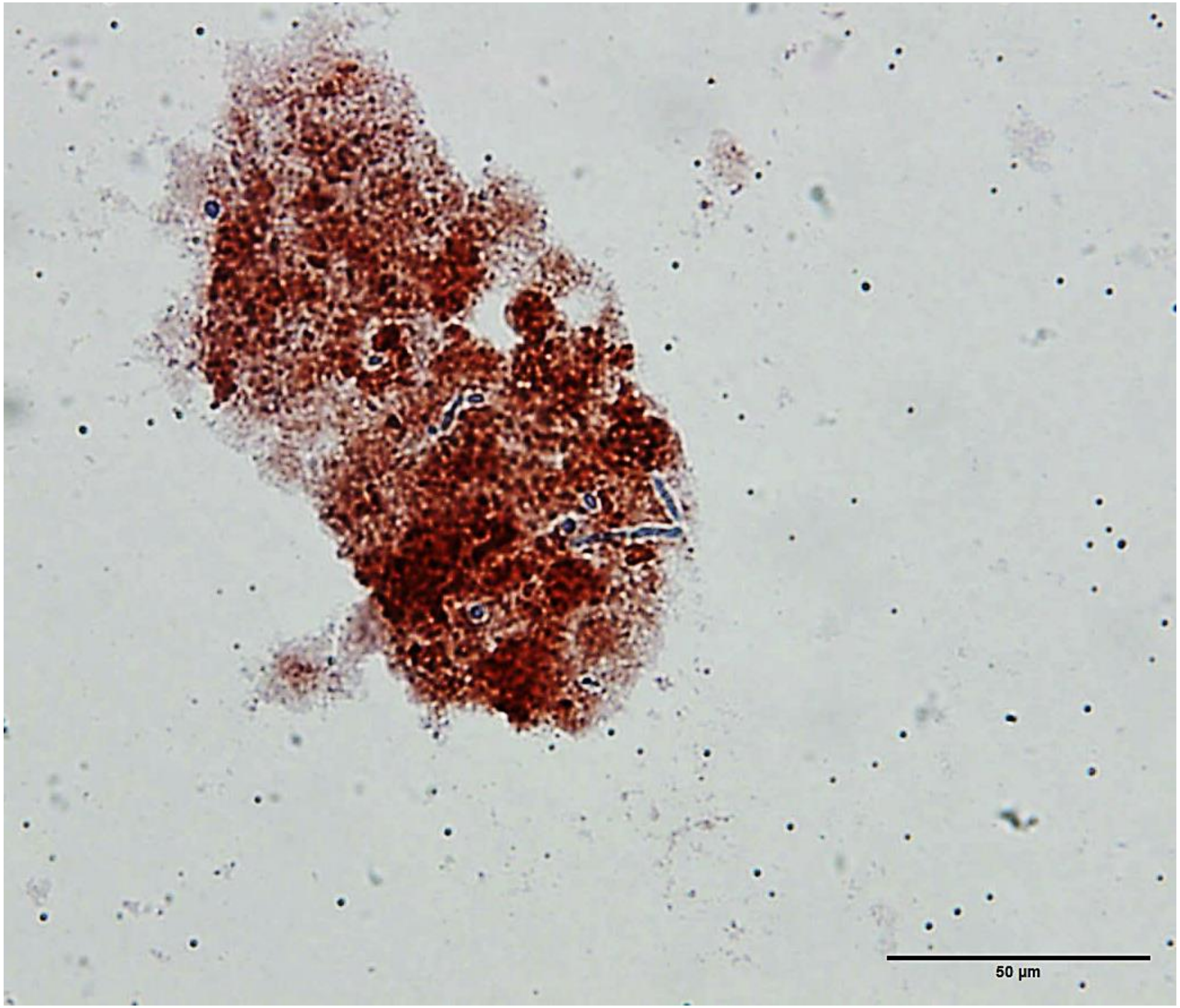

2b) Optical microscopy: urine sample obtained from a 70-year-old female patient who received 4 g of vancomycin over 5 days, developed stage III AKI and displayed smooth vancomycin-specific casts. Peak vancomycin through level: 122 mg/L. Urinary pH: 8.2

2c

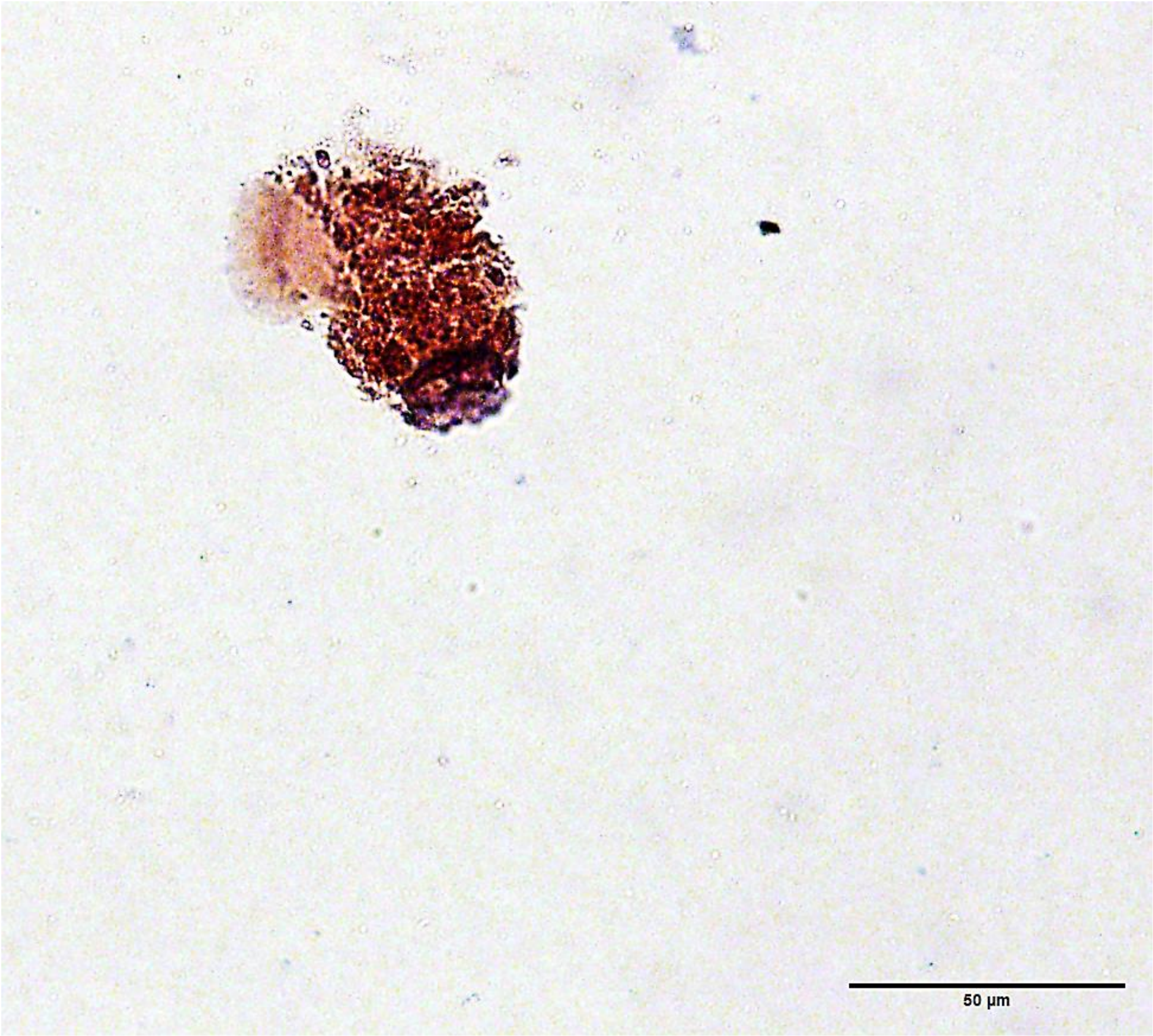

2c) Optical microscopy: urine sample obtained from a 70-year-old female patient who received 2 g of vancomycin over 48 hours, developed stage III acute kidney injury (AKI), and exhibited granular vancomycin-specific casts. Peak vancomycin through level: 61 mg/L. Urinary pH: 5.3

2d

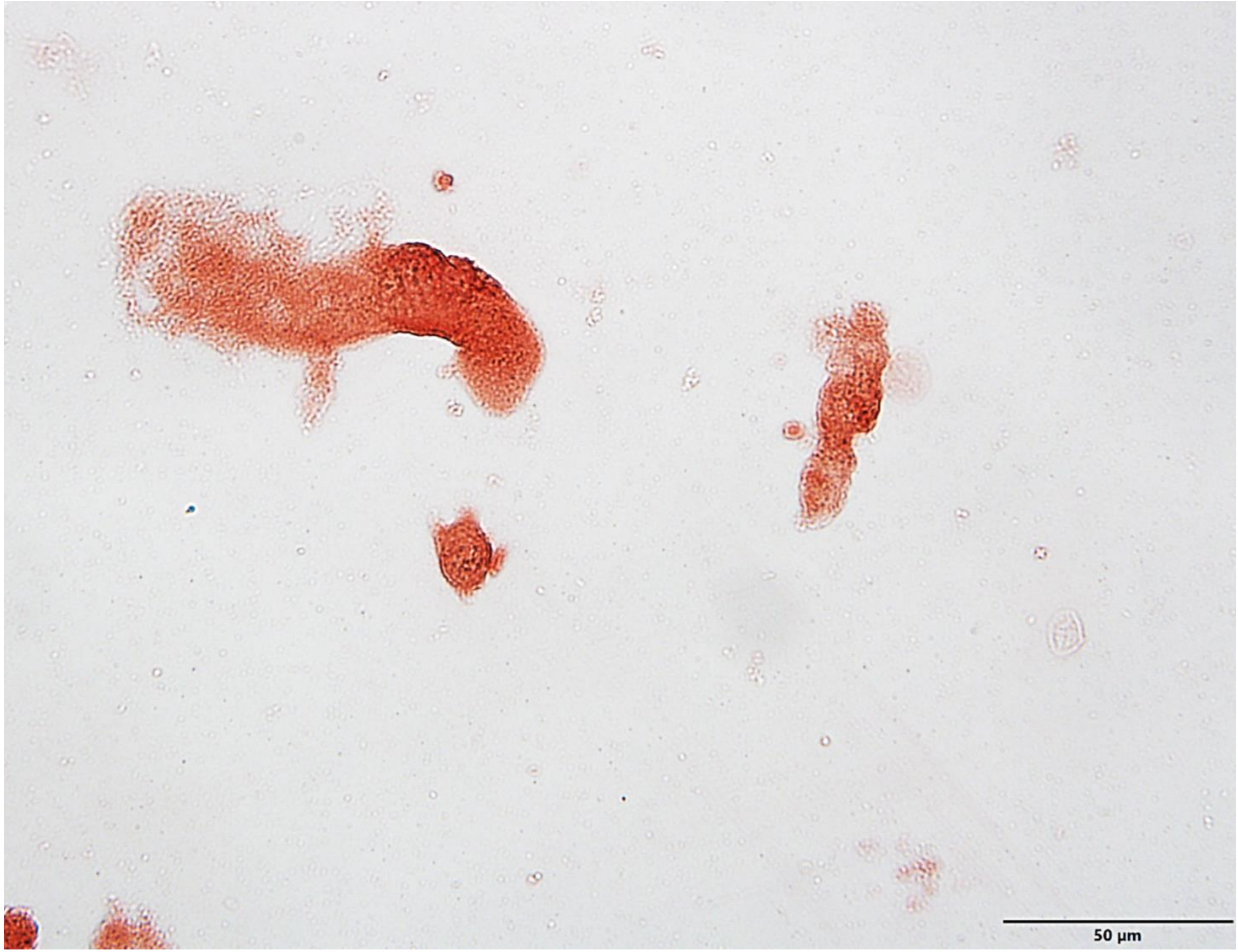

2d) Optical microscopy. Urine sample obtained from a 66-year-old male patient who received 2 g of vancomycin over 2 days, developed stage III AKI and displayed smooth vancomycin-specific casts. Peak vancomycin through level: 43 mg/L. Urinary pH: 6

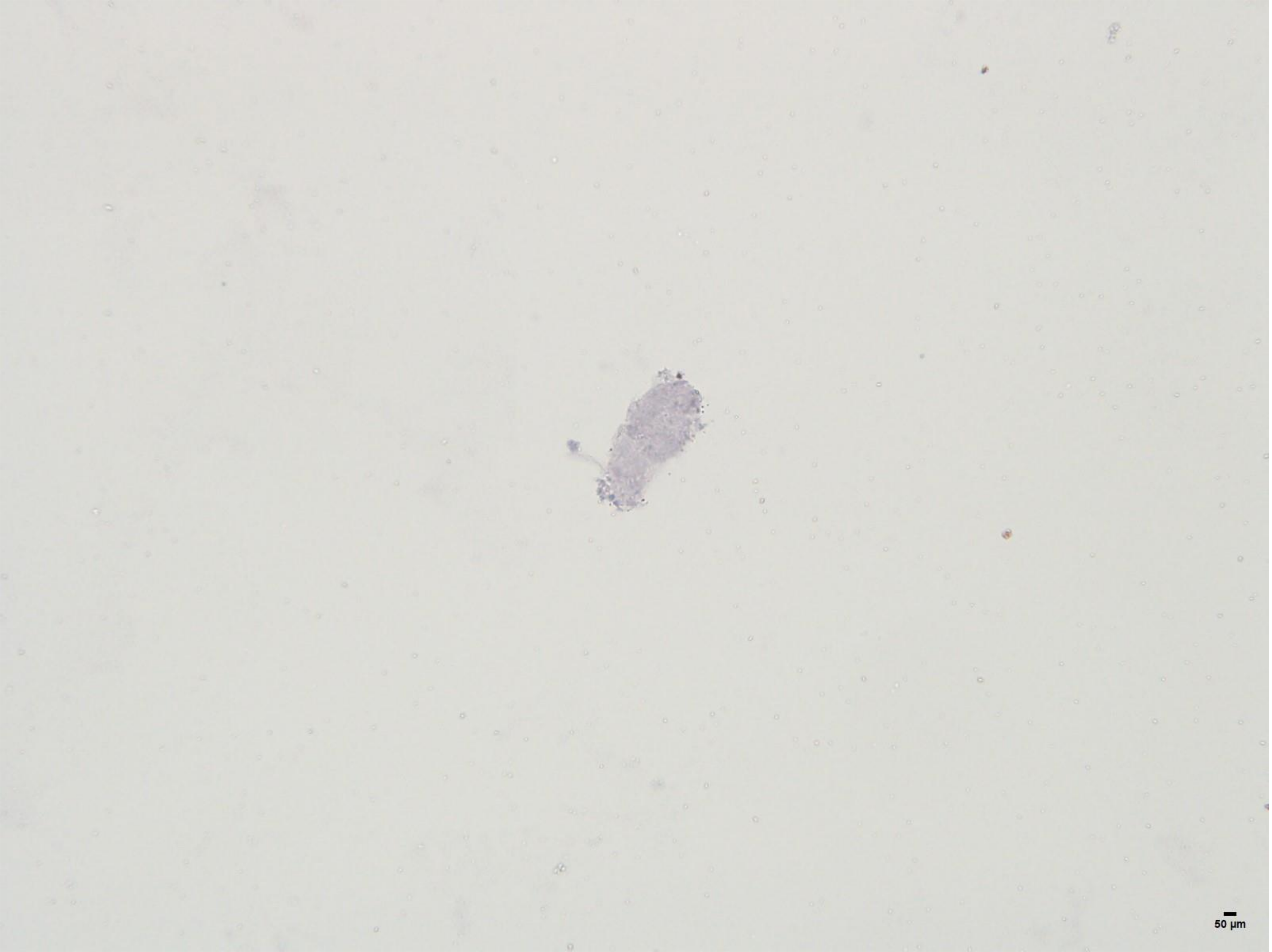
50 µm

2e) Optical microscopy. Urine sample obtained from a 51-year-old male patient who received 1 g of vancomycin over 2 days, in the setting of stage V chronic kidney disease and displayed casts, negative for vancomycin immunostaining. Peak vancomycin through level: 15 mg/L. Urinary pH: 5.9

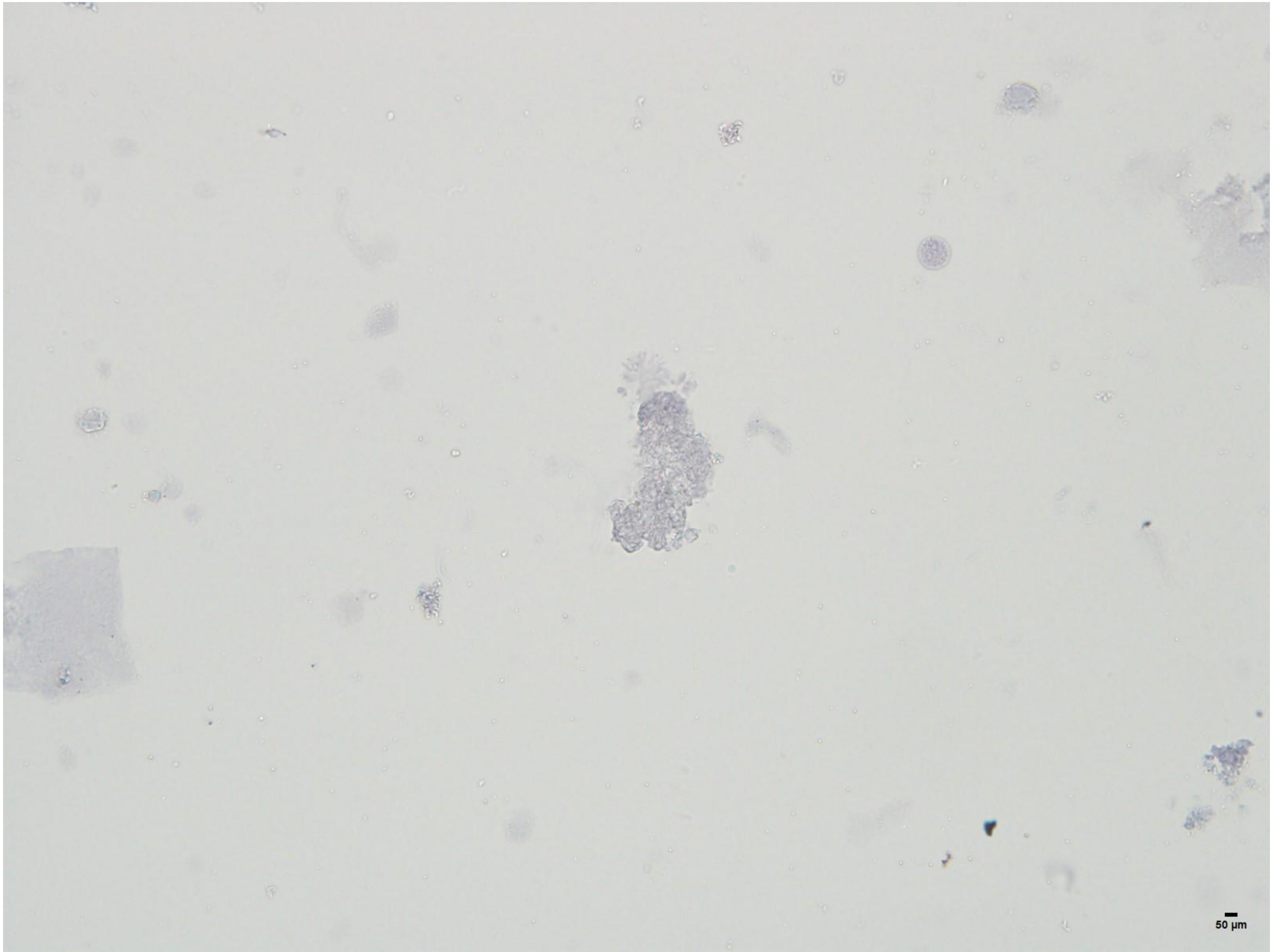
50 µm

2f) Optical microscopy. Urine sample obtained from a 82-year-old male patient who received 10 g of vancomycin over 5 days, in the setting of stage V chronic kidney disease and displayed casts, negative for vancomycin immunostaining. Peak vancomycin through level: 24 mg/L. Urinary pH: 6.2
